# Crystal structure of 1-bromo-2-(phenyl­selen­yl)benzene

**DOI:** 10.1107/S205698901500345X

**Published:** 2015-02-28

**Authors:** Bronte J. Charette, Jamie S. Ritch

**Affiliations:** a Department of Chemistry, The University of Winnipeg, 515 Portage Avenue, Winnipeg, MB, R3B 2E9, Canada; b Department of Chemistry, 360 Parker Building, University of Manitoba, Winnipeg, MB, R3T 2N2, Canada

**Keywords:** crystal structure, π–π inter­actions, organoselenium compounds

## Abstract

In the title compound, C_12_H_9_BrSe, the Se atom exhibits a bent geometry, with a C—Se—C bond angle of 99.19 (6)°. The *ortho* Se and Br atoms are slightly displaced from opposite faces of the mean plane of the benzene ring [by 0.129 (2) and 0.052 (2) Å, respectively]. The planes of the benzene and phenyl rings form a dihedral angle of 72.69 (5)°. In the crystal, π-stacking inter­actions between inversion-related phenyl rings are observed, with a centroid–centroid distance of 3.630 (1) Å.

## Abstract 2

The first crystal structure determination of 1-bromo-2-(phenyl­selen­yl)benzene is presented. The mol­ecules form weak dimers through displaced parallel π-stacking inter­actions.

## Chemical context   

Organoselenium compounds have been found to have diverse scientific applications. For instance, the anti­oxidant capabilities of the gluta­thione peroxidases has inspired the synthesis of selenium-containing enzyme mimetics for therapeutic use (Schewe, 1995 ▸), and examples are known of selenium-based conjugated materials exhibiting superconductivity (Jérome *et al.*, 1980 ▸). Our research group is inter­ested in organoselenium compounds in the context of designing ligands for coordination to transition metals to generate catalytic complexes. This is an area of growing inter­est, as examples of selenium-containing catalysts with higher activity than the ubiquitous phosphine analogues are discovered (Kumar *et al.*, 2012 ▸). The title compound represents a potentially valuable starting material for the synthesis of ligands containing –SePh donor groups, as the *ortho*-Br atom provides a site of functionalization *via*, for example, lithium halogen exchange followed by electrophile addition, or a metal-catalyzed cross-coupling reaction. Though previously prepared (Cristau *et al., 1985 ▸*), its structure has remained unreported.
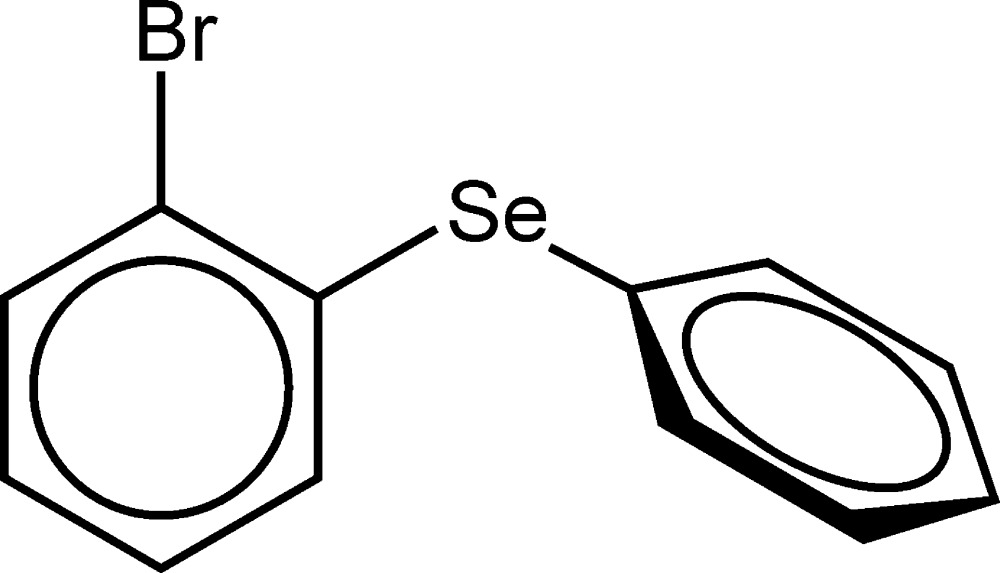


## Structural commentary   

**Figure 1:**
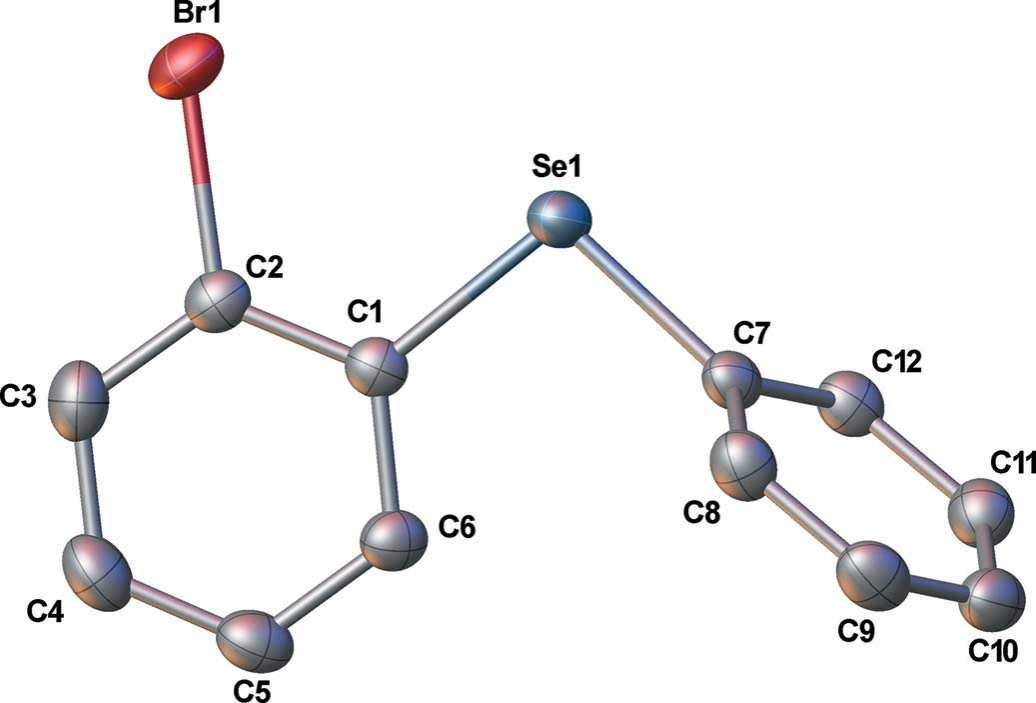
The mol­ecular structure of the title compound, (I), showing 50% probability ellipsoids.

The mol­ecular structure of the title compound, (I), is depicted in Fig. 1 ▸. The asymmetric unit possesses one complete mol­ecule, which features no disorder. The central Se atom exhibits a bent geometry [C1—Se1—C7 = 99.19 (6)°]. The two planes comprising the benzene and phenyl ring C atoms are twisted by 72.69 (5)° relative to each other. The Br and Se atoms are twisted with respect to the disubstituted benzene ring, as evidenced by displacements in opposite directions from the mean plane of the ring by 0.052 (2) and 0.129 (2) Å, respectively, and the torsion angle Br1—C2—C1—Se1 is 4.2 (1)°.

The Se—C distances of 1.9171 (14) and 1.9198 (14) Å are equal within experimental error. At 1.9044 (14) Å, the C—Br distance is measurably shorter than the Se—C bond lengths.

## Supra­molecular features   

**Figure 2:**
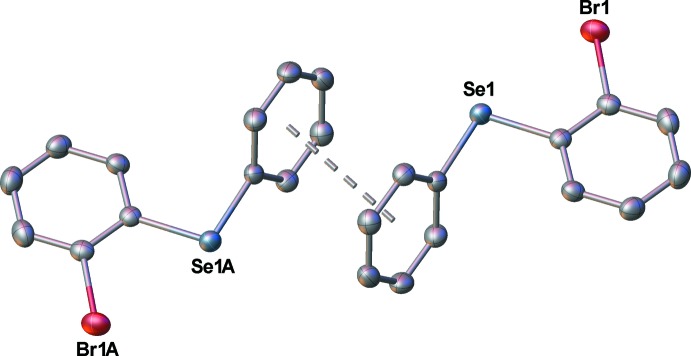
Slipped π-stacked dimers of 1-bromo-2-(phenyl­selen­yl)benzene. Each mol­ecule is related to the other by an inversion centre at the centre of the centroid–centroid line.

**Figure 3:**
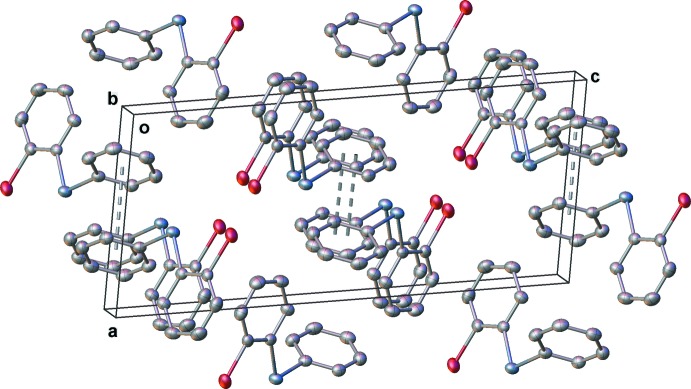
Packing diagram for (I), viewed along the crystallographic *b* axis.

The closest inter­molecular Se⋯Br distance is 3.8013 (3) Å, which lies outside the sum of the van der Waals radii (3.75 Å) for these two elements (Bondi, 1964 ▸). The phenyl group of each mol­ecule is associated with the same group on an adjacent mol­ecule by a slipped π-stacking inter­action (Fig. 2 ▸). The two mol­ecules in the dimeric units are situated about a crystallographic inversion centre. The centroid-to-centroid separation of the aromatic rings is 3.630 (1) Å, while the nearest centroid-to-plane distance is 3.378 (1) Å. Together, these are indicative of the slipped nature of the π–π inter­action. The ring separation is in the normal range (*ca* 3.3–3.8 Å) for π-stacked inter­actions (Janiak, 2000 ▸). The packing is illustrated in Fig. 3 ▸.

## Database survey   

A search of the Cambridge Structural Database (CSD, Version 5.35; Groom & Allen, 2014 ▸) reveals 172 structures featuring two-coordinate aryl-substituted selenium centres. The mean bond angle of 98 (4)° and Se—C(ar­yl) distance of 1.92 (2) Å for these structures match well with the parameters observed for 1-bromo-2-(phenyl­selen­yl)benzene.

Only two structures in the CSD feature the title compound as a substructure: bis­(2-bromo-4,5-di­meth­oxy­phen­yl) selenide (SAKBIP; Schiffling and Klar, 1989 ▸) and 1,4-di­bromo-2,3,5,6-tetra­kis­(phenyl­seleno)­benzene (MUHTOZ; Sato & Kanatomi, 2009 ▸). Both of these compounds exhibit similar twisted orientations of the two aromatic rings, but lack π-stacking secondary bonding inter­actions, presumably due to their highly substituted nature. By contrast, the structure of a less sterically crowded analogue, 1-bromo-8-(phenyl­selen­yl)naph­thalene (CIKPUI; Fuller *et al.*, 2007 ▸), exhibits slipped π-stacking of the naphthalene rings.

## Synthesis and crystallization   

**Figure 4:**
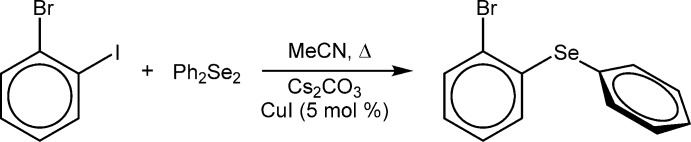
The synthetic route to 1-bromo-2-(phenyl­selen­yl)benzene, (I).

1-Bromo-2-(phenyl­selen­yl)benzene has been prepared in pre­vious reports using several methodologies, including nickel(II)-catalyzed coupling of NaSePh with 1,2-di­bromo­benzene (Cristau *et al.*, 1985 ▸) and the copper-catalyzed coupling of diphenyl diselenide with 1-bromo-2-iodo­benzene (Dandapat *et al.*, 2011 ▸), which is the procedure followed for this study (Fig. 4 ▸). Purification *via* flash column chromatog­raphy with a silica stationary phase was conducted as reported. Though described by Dandapat *et al.* (2011 ▸) as being a ‘slightly brown oil’, we found that this compound was a nearly colourless liquid which slowly crystallized upon standing at room temperature. NMR spectroscopic analysis matched the reported data.

Though quite soluble in common solvents, including nonpolar solvents such as hexa­nes, in the highly lipophilic hexa­methyl­disiloxane we found this substance was only moderately soluble. It crystallized readily as transparent colourless crystals from a solution in this solvent upon storage at 273 K.

## Refinement   

Crystal data, data collection and structure refinement details are summarized in Table 1 ▸. No special considerations were needed for the refinement. H atoms were placed in calculated positions, with C—H = 0.95 Å and *U*_iso_(H) = 1.2*U*_eq_(C), and treated in a riding-model approximation.

## Supplementary Material

Crystal structure: contains datablock(s) I. DOI: 10.1107/S205698901500345X/lh5753sup1.cif

Structure factors: contains datablock(s) I. DOI: 10.1107/S205698901500345X/lh5753Isup2.hkl

Supporting information file. DOI: 10.1107/S205698901500345X/lh5753Isup3.cdx

Supporting information file. DOI: 10.1107/S205698901500345X/lh5753Isup4.cml

CCDC reference: 1050354

Additional supporting information:  crystallographic information; 3D view; checkCIF report

## Figures and Tables

**Table 1:** Experimental details

Crystal data	
Chemical formula	C_12_H_9_BrSe
*M*_r_	312.06
Crystal system, space group	Monoclinic, *P*2_1_/*c*
Temperature (K)	173
*a*, *b*, *c* ()	8.1171(4), 7.6028(4), 18.1345(10)
()	99.2668(6)
*V* (^3^)	1104.52(10)
*Z*	4
Radiation type	Mo *K*
(mm^1^)	6.97
Crystal size (mm)	0.35 0.32 0.26

Data collection	
Diffractometer	Bruker APEXII CCD
Absorption correction	Numerical (*SADABS*; Bruker, 2013 ▸)
*T*_min_, *T*_max_	0.205, 0.361
No. of measured, independent and observed [*I* > 2(*I*)] reflections	21749, 2742, 2528
*R*_int_	0.016
(sin /)_max_ (^1^)	0.669

Refinement	
*R*[*F* ^2^ > 2(*F* ^2^)], *wR*(*F* ^2^), *S*	0.017, 0.042, 1.06
No. of reflections	2742
No. of parameters	127
H-atom treatment	H-atom parameters constrained
_max_, _min_ (e ^3^)	0.28, 0.45

Computer programs: *APEX2* and *SAINT* (Bruker, 2013 ▸), *SHELXS97* (Sheldrick, 2008 ▸), *SHELXL2014* (Sheldrick, 2015 ▸) and *OLEX2* (Dolomanov *et al.*, 2009 ▸).

## supplementary crystallographic information

### Crystal data

**Table:**

C_12_H_9_BrSe	*F*(000) = 600
*M**_r_* = 312.06	*D*_x_ = 1.877 Mg m^−^^3^
Monoclinic, *P*2_1_/*c*	Mo *K*α radiation, λ = 0.71073 Å
*a* = 8.1171 (4) Å	Cell parameters from 9910 reflections
*b* = 7.6028 (4) Å	θ = 2.3–28.2°
*c* = 18.1345 (10) Å	µ = 6.97 mm^−^^1^
β = 99.2668 (6)°	*T* = 173 K
*V* = 1104.52 (10) Å^3^	Fragment, colourless
*Z* = 4	0.35 × 0.32 × 0.26 mm

### Data collection

**Table:**

Bruker APEXII CCD diffractometer	2528 reflections with *I* > 2σ(*I*)
ω scans	*R*_int_ = 0.016
Absorption correction: numerical (*SADABS*; Bruker, 2013)	θ_max_ = 28.4°, θ_min_ = 2.3°
*T*_min_ = 0.205, *T*_max_ = 0.361	*h* = −10→10
21749 measured reflections	*k* = −10→10
2742 independent reflections	*l* = −24→24

### Refinement

**Table:**

Refinement on *F*^2^	Primary atom site location: structure-invariant direct methods
Least-squares matrix: full	Hydrogen site location: inferred from neighbouring sites
*R*[*F*^2^ > 2σ(*F*^2^)] = 0.017	H-atom parameters constrained
*wR*(*F*^2^) = 0.042	*w* = 1/[σ^2^(*F*_o_^2^) + (0.0194*P*)^2^ + 0.5087*P*] where *P* = (*F*_o_^2^ + 2*F*_c_^2^)/3
*S* = 1.06	(Δ/σ)_max_ = 0.001
2742 reflections	Δρ_max_ = 0.28 e Å^−^^3^
127 parameters	Δρ_min_ = −0.45 e Å^−^^3^
0 restraints	

### Special details

**Table:**

Experimental. The following wavelength and cell were deduced by *SADABS* from the direction cosines *etc*. They are given here for emergency use only: *CELL* 0.71074 8.140 7.626 18.183 89.999 99.279 90.004.
Geometry. All e.s.d.'s (except the e.s.d. in the dihedral angle between two l.s. planes) are estimated using the full covariance matrix. The cell e.s.d.'s are taken into account individually in the estimation of e.s.d.'s in distances, angles and torsion angles; correlations between e.s.d.'s in cell parameters are only used when they are defined by crystal symmetry. An approximate (isotropic) treatment of cell e.s.d.'s is used for estimating e.s.d.'s involving l.s. planes.

### Fractional atomic coordinates and isotropic or equivalent isotropic displacement parameters (Å^2^)

**Table:**

	*x*	*y*	*z*	*U*_iso_*/*U*_eq_	
Br1	0.37269 (2)	0.33279 (2)	0.26417 (2)	0.03601 (5)	
Se1	0.39491 (2)	0.65383 (2)	0.38572 (2)	0.02860 (5)	
C1	0.19202 (17)	0.52181 (18)	0.36221 (7)	0.0237 (3)	
C2	0.18210 (18)	0.39017 (19)	0.30840 (8)	0.0266 (3)	
C3	0.0361 (2)	0.2971 (2)	0.28518 (9)	0.0349 (3)	
H3	0.0323	0.2087	0.2479	0.042*	
C4	−0.1043 (2)	0.3346 (2)	0.31696 (10)	0.0384 (4)	
H4	−0.2058	0.2735	0.3008	0.046*	
C5	−0.09621 (19)	0.4610 (2)	0.37222 (9)	0.0341 (3)	
H5	−0.1918	0.4846	0.3947	0.041*	
C6	0.05039 (18)	0.55397 (19)	0.39518 (8)	0.0283 (3)	
H6	0.0546	0.6399	0.4335	0.034*	
C7	0.32416 (18)	0.82409 (18)	0.45228 (8)	0.0255 (3)	
C8	0.22331 (19)	0.9649 (2)	0.42396 (9)	0.0315 (3)	
H8	0.1849	0.9747	0.3718	0.038*	
C9	0.17967 (19)	1.0906 (2)	0.47282 (10)	0.0350 (3)	
H9	0.1097	1.1861	0.4541	0.042*	
C10	0.23779 (19)	1.0773 (2)	0.54880 (10)	0.0343 (3)	
H10	0.2074	1.1636	0.5820	0.041*	
C11	0.3399 (2)	0.9387 (2)	0.57634 (9)	0.0325 (3)	
H11	0.3809	0.9311	0.6283	0.039*	
C12	0.38282 (18)	0.81058 (19)	0.52816 (9)	0.0283 (3)	
H12	0.4518	0.7145	0.5471	0.034*	

### Atomic displacement parameters (Å^2^)

**Table:**

	*U*^11^	*U*^22^	*U*^33^	*U*^12^	*U*^13^	*U*^23^
Br1	0.03941 (9)	0.04133 (10)	0.02890 (8)	0.00838 (7)	0.01038 (6)	−0.00290 (6)
Se1	0.02379 (8)	0.02797 (8)	0.03495 (9)	−0.00057 (5)	0.00750 (6)	−0.00292 (6)
C1	0.0245 (6)	0.0210 (6)	0.0253 (6)	0.0015 (5)	0.0034 (5)	0.0045 (5)
C2	0.0294 (7)	0.0261 (7)	0.0244 (6)	0.0041 (6)	0.0045 (5)	0.0030 (5)
C3	0.0418 (9)	0.0299 (8)	0.0310 (8)	−0.0032 (7)	−0.0002 (6)	−0.0033 (6)
C4	0.0322 (8)	0.0352 (9)	0.0460 (9)	−0.0086 (7)	0.0010 (7)	0.0016 (7)
C5	0.0274 (7)	0.0312 (8)	0.0449 (9)	−0.0010 (6)	0.0098 (6)	0.0047 (7)
C6	0.0292 (7)	0.0230 (7)	0.0340 (7)	0.0014 (6)	0.0089 (6)	0.0009 (6)
C7	0.0228 (6)	0.0208 (6)	0.0332 (7)	−0.0020 (5)	0.0049 (5)	−0.0010 (5)
C8	0.0296 (7)	0.0279 (7)	0.0350 (8)	0.0009 (6)	−0.0004 (6)	0.0023 (6)
C9	0.0281 (7)	0.0251 (7)	0.0504 (9)	0.0042 (6)	0.0023 (7)	0.0019 (7)
C10	0.0305 (8)	0.0280 (8)	0.0457 (9)	−0.0021 (6)	0.0105 (7)	−0.0069 (7)
C11	0.0338 (8)	0.0325 (8)	0.0313 (7)	−0.0031 (6)	0.0057 (6)	−0.0008 (6)
C12	0.0274 (7)	0.0233 (7)	0.0339 (7)	−0.0006 (5)	0.0038 (6)	0.0047 (6)

### Geometric parameters (Å, º)

**Table:**

Br1—C2	1.9044 (14)	C6—H6	0.9500
Se1—C1	1.9171 (14)	C7—C8	1.395 (2)
Se1—C7	1.9198 (14)	C7—C12	1.386 (2)
C1—C2	1.391 (2)	C8—H8	0.9500
C1—C6	1.4001 (19)	C8—C9	1.387 (2)
C2—C3	1.386 (2)	C9—H9	0.9500
C3—H3	0.9500	C9—C10	1.386 (2)
C3—C4	1.387 (2)	C10—H10	0.9500
C4—H4	0.9500	C10—C11	1.384 (2)
C4—C5	1.383 (2)	C11—H11	0.9500
C5—H5	0.9500	C11—C12	1.390 (2)
C5—C6	1.389 (2)	C12—H12	0.9500
			
C1—Se1—C7	99.19 (6)	C8—C7—Se1	120.23 (11)
C2—C1—Se1	118.90 (10)	C12—C7—Se1	118.99 (11)
C2—C1—C6	117.80 (13)	C12—C7—C8	120.67 (14)
C6—C1—Se1	123.27 (11)	C7—C8—H8	120.4
C1—C2—Br1	120.07 (11)	C9—C8—C7	119.25 (14)
C3—C2—Br1	117.89 (11)	C9—C8—H8	120.4
C3—C2—C1	122.04 (14)	C8—C9—H9	119.9
C2—C3—H3	120.4	C10—C9—C8	120.25 (15)
C2—C3—C4	119.24 (15)	C10—C9—H9	119.9
C4—C3—H3	120.4	C9—C10—H10	119.9
C3—C4—H4	120.1	C11—C10—C9	120.16 (15)
C5—C4—C3	119.82 (15)	C11—C10—H10	119.9
C5—C4—H4	120.1	C10—C11—H11	119.9
C4—C5—H5	119.7	C10—C11—C12	120.22 (15)
C4—C5—C6	120.64 (15)	C12—C11—H11	119.9
C6—C5—H5	119.7	C7—C12—C11	119.45 (14)
C1—C6—H6	119.8	C7—C12—H12	120.3
C5—C6—C1	120.39 (14)	C11—C12—H12	120.3
C5—C6—H6	119.8		
			
Br1—C2—C3—C4	179.31 (12)	C4—C5—C6—C1	0.5 (2)
Se1—C1—C2—Br1	4.17 (16)	C6—C1—C2—Br1	−177.44 (10)
Se1—C1—C2—C3	−175.83 (12)	C6—C1—C2—C3	2.6 (2)
Se1—C1—C6—C5	175.87 (11)	C7—C8—C9—C10	0.9 (2)
Se1—C7—C8—C9	−177.21 (12)	C8—C7—C12—C11	0.2 (2)
Se1—C7—C12—C11	176.40 (11)	C8—C9—C10—C11	0.1 (2)
C1—C2—C3—C4	−0.7 (2)	C9—C10—C11—C12	−1.0 (2)
C2—C1—C6—C5	−2.4 (2)	C10—C11—C12—C7	0.8 (2)
C2—C3—C4—C5	−1.3 (2)	C12—C7—C8—C9	−1.1 (2)
C3—C4—C5—C6	1.4 (3)		
